# Preoperative regional cerebral oxygen saturation is a predictor of postoperative delirium in on-pump cardiac surgery patients: a prospective observational trial

**DOI:** 10.1186/cc10454

**Published:** 2011-09-19

**Authors:** Julika Schoen, Joscha Meyerrose, Hauke Paarmann, Matthias Heringlake, Michael Hueppe, Klaus-Ulrich Berger

**Affiliations:** 1 Department of Anaesthesiology, University of Luebeck, Ratzeburger Allee 160, Luebeck, 23538, Germany

## Abstract

**Introduction:**

Postoperative delirium is an important problem in patients undergoing major surgery. Cerebral oximetry is a non-invasive method to detect imbalances in the cerebral oxygen supply/demand-ratio. Low preoperative cerebral oxygen saturation (ScO_2_) levels have been associated with postoperative delirium in non-cardiac surgery patients. The present prospective observational study determines the relationship between pre- and intra-operative ScO_2 _levels and postoperative delirium in patients undergoing on-pump cardiac surgery.

**Methods:**

After approval of the local ethical committee and written informed consent, *N *= 231 patients scheduled for elective/urgent cardiac surgery were enrolled. Delirium was assessed by the confusion-assessment-method for the intensive care unit (CAM-ICU) on the first three days after surgery. ScO_2 _was obtained on the day before surgery, immediately before surgery and throughout the surgical procedure. Preoperative cognitive function, demographic, surgery related, and intra- and post-operative physiological data were registered.

**Results:**

Patients with delirium had lower pre- and intra-operative ScO_2 _readings, were older, had lower mini-mental-status-examination(MMSE) scores, higher additive EuroScore and lower preoperative haemoglobin-levels. The binary logistic regression identified older age, lower MMSE, neurological or psychiatric disease and lower preoperative ScO_2 _as independent predictors of postoperative delirium.

**Conclusions:**

The presented study shows that a low preoperative ScO_2 _is associated with postoperative delirium after on-pump cardiac surgery.

## Introduction

Delirium is a common and serious problem in critically ill patients [1] and occurs in up to 41% [2] of cardiac surgical patients. Several preoperative risk factors for delirium after cardiac surgery have been identified: age, cognitive status, the severity of illness (that is, cardiogenic or septic shock), anemia, and certain medications like sedatives, anti-cholinergics, or hypnotics [2-6].

Whereas the hyperactive form of delirium is easily recognized, the much more frequent hypoactive form is often missed by nurses and intensive care physicians [7,8]. As both the hyperactive and the hypoactive form of delirium have been shown to be associated with prolonged hospital stay, increased mortality, functional decline, and long-term cognitive impairment [1,9-12], specific diagnostic algorithms need to be implemented and prevention of delirium should be the focus of perioperative cardiac surgery care [13].

Near-infrared spectroscopy offers the possibility of measuring the oxygen supply/demand ratio in frontal brain tissue non-invasively [14,15]. We recently showed that preoperative cerebral oxygen saturation determined by near-infrared spectroscopy is reflective of the severity of cardiopulmonary dysfunction and is associated with postoperative morbidity and mortality in patients undergoing on-pump cardiac surgery [16]. As the preoperative severity of illness has been shown to be a predictor of postoperative delirium [2], an association between preoperative cerebral oxygenation and postoperative delirium is conceivable. In a small cohort of non-cardiac surgical patients, Morimoto and colleagues [17] could already identify preoperative cerebral saturation to be an independent risk factor for postoperative delirium.

The present observational cohort study was designed to explore the relationship between pre- and intraoperative cerebral oxygen saturation levels and postoperative delirium in patients undergoing on-pump cardiac surgery. The primary hypothesis was that patients who develop postoperative delirium differ from those who do not develop delirium after cardiac surgery in preoperative regional cerebral oxygen saturation. The secondary hypothesis was that pre- and intraoperative cerebral oxygen saturation (ScO_2_) values influence the risk of postoperative delirium in patients undergoing on-pump cardiac surgery. The present work analyzes a subset of patients of our previous study [16] who underwent additional specific testing on delirium.

## Materials and methods

The present report is a substudy of a large observational study that our group published earlier this year [16] and has been approved by the local ethics committee (Ethikkommission der Universität zu Lübeck, Lübeck, Germany). After written informed consent was obtained, 256 consecutive patients were enrolled in this prospective observational study during a 3-month period (1 October 2008 to 31 January 2009). The inclusion criteria were elective or urgent cardiac surgery and ability to perform the confusion assessment method for the intensive care unit (CAM-ICU). Exclusion criteria were age below 18 years, hemodynamic instability with emergency indication for cardiac surgery, or insufficient knowledge of the German language.

The 168 patients who were enrolled between 1 October and 31 December 2008 were participants of the outcome study that our working group published earlier this year [16]. That study included 1,178 patients in total, so the present study analyzes almost 15% of the patients in the outcome study.

On the day before surgery, after inclusion and written informed consent in the observational study, patients were assessed with the CAM-ICU [18] in order to affirm the inclusion criteria for the substudy. The Mini-Mental Status Examination (MMSE) was conducted to record preoperative cognitive function. Demographic and physiological data as well as preoperative morbidity and medication were documented. Routine blood tests included leukocytes, C-reactive protein, hemoglobin, creatinine, and N-terminal pro B-type natriuretic peptide (NTproBNP) electrochemiluminescence immunoassay (Elecsys proBNP sandwich immunoassay; Grenzach-Wyhlen, Germany, Roche Diagnostics).

Regional cerebral oxygen saturation (ScO_2_) measurement was performed by using the INVOS Cerebral Oximeter 5100 (Somanetics, Troy, MI, USA) with bi-hemispherical near-infrared spectroscopy sensors. The mean of both hemispheres was used for analysis. ScO_2 _measurement was performed on the day before surgery. The first value (ScO_2_room) was obtained in a sitting position without supplemental oxygen. To correct for possible hypoxemia, the patients were supplied with 4 L per minute supplemental oxygen until peripheral arterial oxygen saturation (SaO_2_) reached greater than 98%. After stabilization of the signal (2 to 4 minutes), the ScO_2 _after compensation of hypoxemia was determined (ScO_2_ox).

Anesthesia was performed in accordance with institutional standards with etomidate 0.2 mg/kg, sufentanil 0.5 μg/kg, and rocuroniumbromide 0.7 mg/kg for induction and remifentanil 0.25 μg/kg per minute and sevoflurane 1.0 to 1.5 minimum alveolar concentration (MAC) for maintenance of anesthesia. Hemodynamic monitoring was applied in accordance with institutional standards. Surgery was performed in mild hypothermia by using antegrade blood cardioplegia.

All patients were monitored with continuous bi-hemispherical cerebral oxygen saturation monitoring in addition to the standard monitoring including electrocardiogram, SaO_2_, invasive blood pressure, and central venous pressure. Cerebral oximetry was part of the routine monitoring, and interventions were made by the attending anesthetist as required. No specific algorithm has been applied. The institutional recommendation includes head repositioning, control and optimization of carbon dioxide, and optimization of mean arterial pressure as first-line interventions and reevaluation and optimization of cardiac performance (or pump flow on cardiopulmonary bypass) and reevaluation of transfusion trigger as second-line interventions.

Measurement was recorded and analyzed after cessation of the study period. Intraoperative baseline, lowest intraoperative values as well as areas under the curve (AUCs) resulting from the cutoff values 'below 80% of intraoperative baseline' and 'below ScO_2 _= 50% absolute value' were identified and documented.

Postoperative sedation followed the institutional standard and was performed with starting doses of remifentanil 0.125 μg/kg per minute and propofol 1.5 to 2 mg/kg per hour. In the absence of contraindications, a continuous application of metamizol 3 g per 24 hours was started. After adequate rewarming, the propofol dose was stopped, single doses of pethidin 25 mg and piritramid 3.75 mg were applied, and the remifentanil dose was continuously reduced. Further piritramid was applied according to patients' analgesic needs.

To determine possible intra- and postoperative differences in the groups, intraoperative requirement of packed red cells, fresh frozen plasma, and inotropic drugs was documented. Postoperative markers of myocardial injury, inflammation, and renal function were documented.

### Determination of primary endpoint

On days 1, 2, and 3 after surgery, patients were assessed for delirium with the CAM-ICU. The CAM-ICU includes the four features of 'acute onset or fluctuating course', 'inattention', 'disorganized thinking', and 'altered level of consciousness'. The positive diagnosis 'delirium' affords a positive result in the first two features and either feature three or four. The determination of sedation status with the Richmond Agitation Sedation Scale (RASS) [19] is required for assessment of testability of the sedated patient and for differentiation of hypo- and hyperactive delirium. The diagnosis of delirium requires an RASS score of at least -3. A positive diagnosis of delirium with RASS score of not more than -1 is defined as the hypoactive form of delirium, and a positive diagnosis with RASS score of greater than 0 is defined as hyperactive delirium. The CAM-ICU was performed by a trained investigator who was blinded to the intraoperative ScO_2 _measurements. A positive CAM-ICU in at least one of the first three postoperative days was defined as 'delirium'.

### Statistical analysis

Statistical analyses were performed with the PASW Statistics 18 (formerly SPSS) for Windows (SPSS, Inc., Chicago, IL, USA). After analysis by the Kolmogorov-Smirnov test for normality of distribution, group differences were analyzed by the Student *t *test for independent samples. If normal distribution was not given, the chi-square test (nominal data) or the Mann-Whitney *U *test (continuous data) was used as appropriate.

All preoperative variables differing significantly with a *P *value of less than 0.01 in the univariate analysis were included in a multivariate logistic regression analysis. The best cutoff values for the continuous variables were further determined by receiver operating characteristic (ROC) analysis. Statistical significance was assessed at the 5% level (*P *< 0.05 is statistically significant).

In a *post hoc *power analysis, the prevalence of delirium in the studied sample is sufficient to identify group differences with medium effect size (d = 0.42) with a probability of error of 5% and a power of 80%. A medium effect can be regarded as clinically relevant and we therefore considered the group size to be sufficient for relevant results.

## Results

### Recruitment

**Figure 1:**
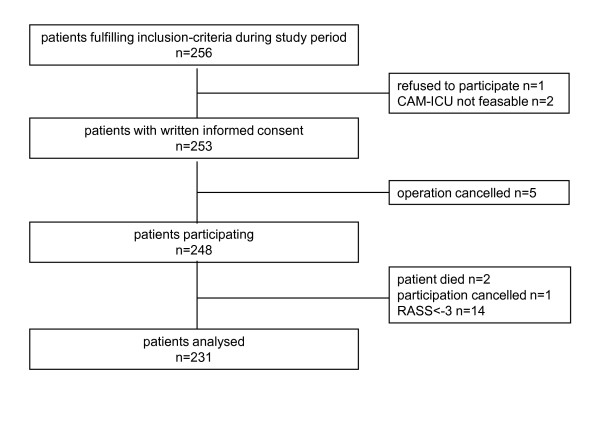
**Recruitment of patients during the 3-month study period**. CAM-ICU, confusion assessment method for the intensive care unit; RASS, Richmond Agitation Sedation Scale.

Two hundred fifty-six patients fulfilled the inclusion criteria during the study period, one patient did not give consent, and in five patients surgery was cancelled. Two patients could not be assessed with the CAM-ICU, one because of severe dementia and the other because of congenital deafness. Seventeen patients had to be excluded from the study postoperatively. Fourteen patients could not be assessed with CAM-ICU, because of deep sedation during the study period (RASS score of less than -3), two patients died, and one patient refused to participate postoperatively, leaving 231 patients for analysis (Figure 1).

### Incidence of delirium

Sixty-two patients (26.8%) developed postoperative delirium. In 45 (72.6%) cases, delirium developed on day 1, in 16 (25.8%) on day 2, and in 1 case (1.6%) on day 3 after surgery. The hypoactive form of delirium was present in 69.4% of cases.

### Univariate analysis

#### ScO_2 _measurements

Patients with delirium showed significantly lower ScO_2_room (without oxygen) and ScO_2_ox (with supplemental oxygen) on the day before surgery (Table 1). The respective peripheral SaO_2 _values on the day before surgery did not differ significantly between patients with and without delirium. Likewise, the ScO_2 _values obtained immediately before induction of anesthesia (ScO_2_ind) were lower in the group of patients who developed delirium. Intraoperatively, the AUCs with the cutoff value below 80% of baseline value did not differ between the groups, whereas the AUC below the absolute value ScO_2 _of less than 50% was larger in the delirium group (Table 1). Furthermore, the minimal intraoperative ScO_2 _(ScO_2_min) was lower in the delirium group, whereas the difference between ScO_2_ox and ScO_2_min (delta ScO_2_ox) did not differ between the groups (Table 1).

**Table 1:** Characterizing measures of preoperative cerebral and arterial oxygen saturation and intraoperative cerebral oxygen saturation in patients with or without postoperative delirium

Parameter	Delirium *n *= 62	Control *n *= 169	*P *value
ScO_2_room	58.1 (7.7)	63.1 (7.2)	≤ 0.001^a^
SaO_2_room	96.5 (1.9)	96.1 (2.0)	0.175^a^
ScO_2_base	62.8 (7.8)	67.6 (6.9)	≤ 0.001^a^
SaO_2_base	99.5 (0.7)	99.4 (0.9)	0.539^a^
ScO_2_base - ScO_2_room	4.6 (3.9)	4.4 (2.8)	0.663^a^
SaO_2_base - SaO_2_room	3.5 (1.8)	2.9 (1.6)	0.053^a^
ScO_2_ind	57.6 (7.5)	63.1 (7.4)	≤ 0.001^a^
Intraoperative ScO_2_			
Lowest value, ScO_2_min	48.6 (9.3)	55.1 (8.6)	≤ 0.001^b^
Highest value	77.4 (6.9)	81.4 (6.4)	≤ 0.001^b^
< 80% of baseline, minimum	3.5 (12.2)	3.9 (15.2)	0.183^b^
< 80% of baseline, AUC	17.2 (48.5)	22.8 (93.9)	0.095^b^
< 50% absolute value, minimum	9.4 (23.3)	3.0 (11.8)	≤ 0.001^b^
< 50% absolute value, AUC	41.6 (114.9)	19.5 (94.9)	≤ 0.001^b^
ScO_2_base - ScO_2_min	13.6 (10.0)	12.6 (8.4)	0.540^a^
ScO_2_room - ScO_2_min	9.1 (9.8)	7.9 (7.7)	0.428^a^

Data other than *P *values are presented as mean (standard deviation). ^a^Student *t *test; ^b^Mann-Whitney *U *test. AUC, area under the curve; SaO_2_base, arterial oxygen saturation with patient breathing 4 L per minute supplemental oxygen; SaO_2_room, arterial oxygen saturation with patient breathing room air; ScO_2_, cerebral oxygen saturation; ScO_2_ind, regional cerebral oxygen saturation immediately before induction of anesthesia; ScO_2_base, regional cerebral oxygen saturation with patient breathing 4 L per minute supplemental oxygen; ScO_2_room, regional cerebral oxygen saturation with patient breathing room air.

**Figure 2:**
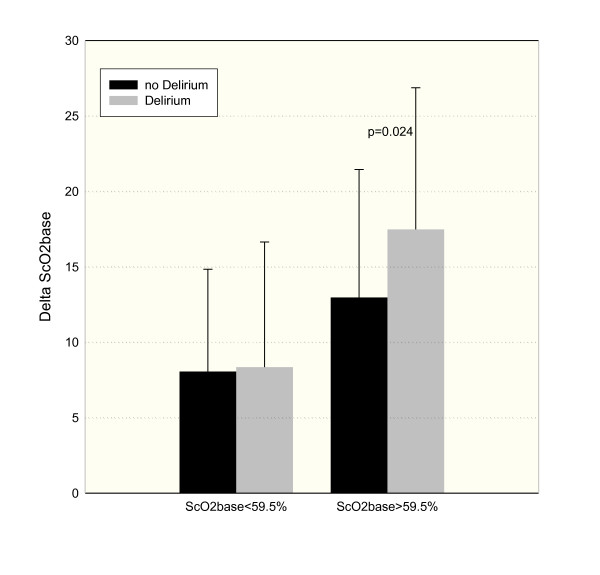
**Intraoperative changes in ScO_2 _in patients with or without delirium classified by normal or low preoperative ScO_2_**. Delta ScO_2_base, difference between preoperative regional cerebral oxygen saturation with oxygen supplementation and minimal intraoperative regional cerebral oxygen saturation; ScO_2_base, regional cerebral oxygen saturation with supplemental oxygen.

For further analysis of the ScO_2 _measurement, we compared the impact of delta ScO_2 _on delirium in patients who started with normal ScO_2 _(ScO_2_ox of greater than 59.5%) and in patients who started with low ScO_2 _(ScO_2_ox of not more than 59.5%). The result is shown in Figure 2. In patients who started with low ScO_2_ox, the groups with and without delirium did not differ in delta ScO_2_ox. But in patients who started at a normal level of ScO_2_ox, those patients who developed delirium had larger intraoperative drops in ScO_2_. The positive predictive value for ScO_2_ox of not more than 59.5% on delirium was 0.56, and the negative predictive value was 0.80.

#### Demographic, neurocognitive, pre- and intraoperative, and laboratory variables

Univariate comparison of preoperative factors between the groups with and without delirium is shown in Table 2. Patients with postoperative delirium were older and had a lower educational level and lower scores in MMSE. Furthermore, patients with delirium had a higher additive EuroScore and suffered more often from diabetes, dementia, or other neurological or psychiatric diseases. These patients had lower hemoglobin and higher creatinine and NTproBNP levels before surgery.

**Table 2:** Preoperative demographics, patient- and surgery-related factors, and laboratory variables in patients with or without delirium

Parameter	Delirium *n *= 62	Control *n *= 169	*P *value
Sex, number (percentage)			
Male	34 (54.8)	115 (68.0)	0.063^a^
Female	28 (45.2)	54 (32.0)	
Age in years, mean (SD)	73.1 (6.7)	64.9 (13.3)	≤ 0.001^b^
Body mass index in kg/m^2^, mean (SD)	27.4 (4.3)	27.6 (4.9)	0.782^b^
Educational level, number (percentage)			
No graduation	7 (11.3)	4 (2.4)	≤ 0.001^a^
Compulsory school	43 (69.4)	81 (47.9)	
Secondary school	10 (16.1)	55 (32.5)	
Vocational diploma	0 (0.0)	7 (4.1)	
General qualification for university entrance	2 (3.2)	22 (13.0)	
MMSE score, mean (SD)	25.32 (3.56)	27.56 (2.25)	≤ 0.001^b^
EuroScore score, mean (SD)	7.9 (3.7)	5.9 (3.5)	≤ 0.001^b^
LVEF, number (percentage)			
< 30%	4 (6.5)	9 (5.4)	0.235^a^
30%-50%	14 (22.6)	23 (13.7)	
> 50%	44 (71.0)	136 (75.6)	
Preoperative morbidity, number (percentage)			
Diabetes mellitus	23 (37.1)	37 (21.9)	0.020^a^
Cerebral vessel stenosis > 50%	8 (12.9)	9 (5.3)	0.051^a^
Dementia	2 (3.2)	0 (0.0)	0.019^a^
Neurological or psychiatric disease	17 (27.4)	12 (7.1)	≤ 0.001^a^
Surgical procedure, number (percentage)			
Valve replacement/repair	18 (29.0)	38 (22.5)	0.201^a^
Coronary artery bypass grafting	31 (50.0)	85 (50.3)	
Combined procedure	7 (11.3)	12 (7.1)	
Other	6 (9.7)	34 (20.1)	
Preoperative laboratory results, mean (SD)			
C-reaktive protein, mg/dL	15.6 (25.4)	10.2 (19.4)	0.149^b^
Leukocytes, /μL	7,408 (2,452)	7,696 (2,141)	0.396^b^
Hemoglobin, g/L	127.1 (17.8)	134.9 (16.1)	0.003^b^
Creatinine, μmol/L	120.2 (120.7)	85.6 (28.8)	0.033^b^
NTproBNP, pg/mL	4,164 (10,127)	1,301 (2,399)	0.001^c^

^a^Chi-square test; ^b^Student *t *test; ^c^Mann-Whitney *U *test. LVEF, left ventricular ejection fraction; MMSE, Mini-Mental Status Examination; NTproBNP, N-terminal pro B-type natriuretic peptide; SD, standard deviation.

Univariate comparison of the intraoperative and postoperative variables is shown in Table 3. The groups were comparable with respect to duration of operation, cardiopulmonary bypass, and aortic crossclamp. Patients who developed delirium had comparable lowest intraoperative hemoglobin but received more packed red cells and more inotropic drugs. In the postoperative course, there were no group differences regarding markers of myocardial injury or inflammation. Patients with delirium developed higher creatinine values that led more often to renal replacement therapy on postoperative day 3. More patients with delirium needed mechanical cardiac support with intra-aortic balloon pump or reintubation. Patients with delirium had a longer duration of mechanical ventilation and a longer length of stay in the ICU.

**Table 3:** Characterizing intraoperative and postoperative data in patients with or without postoperative delirium

Parameter	Delirium *n *= 62	Control *n *= 169	*P *value
Duration of CPB in minutes, mean (SD)	123.0 (53.2)	118.2 (48.6)	0.519^a^
Duration of aortic crossclamp in minutes, mean (SD)	92.8 (40.3)	90.1 (39.3)	0.650^a^
Minimal intraoperative hemoglobin in g/L, mean (SD)	80.3 (10.2)	83.2 (10.8)	0.068^a^
Intraoperative variables in mL, mean (SD)			
Packed red cells	625 (600)	325 (425)	≤ 0.001^a^
Fresh frozen plasma	200 (475)	75 (350)	0.107^a^
Thrombozytes	50 (150)	25 (125)	0.185^a^
Intraoperative cumulative variables in mg, mean (SD)			
Dobutamine	37.8 (38.6)	24.5 (35.1)	0.014^a^
Norepinephrine	0.96 (1.1)	0.45 (0.7)	0.003 ^a^
Milrinone	1.39 (2.0)	0.69 (1.4)	0.011^a^
Marker of myocardial injury, mean (SD)			
MB fraction of CK percentage on POD 1	5.0 (2.7)	4.7 (2.6)	0.457^a^
MB fraction of CK percentage on POD 2	4.0 (3.7)	3.4 (2.7)	0.208^a^
MB fraction of CK percentage on POD 3	4.1 (3.4)	3.9 (3.3)	0.629^a^
Markers of inflammation, mean (SD)			
CRP in mg/dL on POD 1	90.0 (54.8)	83.6 (46.8)	0.483^a^
CRP in mg/dL on POD 2	230.2 (60.5)	218.6 (59.4)	0.214^a^
CRP in mg/dL on POD 3	219.0 (60.8)	204.4 (65.3)	0.159^a^
Leukocytes in μL^-1 ^on POD 1	10,088 (3,833)	10,335 (3,563)	0.651^a^
Leukocytes in μL^-1 ^on POD 2	11,558 (4,056)	11,358 (3,140)	0.731^a^
Leukocytes in μL^-1 ^on POD 3	10,817 (4,386)	10,179 (2,908)	0.321^a^
Marker of renal function, mean (SD)			
Creatinine in μmol/L on POD 1	111.6 (91.2)	87.4 (29.1)	0.046^a^
Creatinine in μmol/L on POD 2	114.8 (49.7)	91.2 (40.9)	0.002^a^
Creatinine in μmol/L on POD 3	111.9 (52.2)	94.7 (48.7)	0.032^a^
Renal replacement therapy, number (percentage)			
POD 1	4 (6.5)	3 (1.8)	0.068 ^b^
POD 2	4 (6.5)	3 (1.8)	0.068^b^
POD 3	4 (6.5)	2 (1.2)	0.030^b^
Intra-aortic balloon pump, number (percentage)			
POD 1	6 (9.7)	3 (1.8)	0.006 ^b^
POD 2	4 (6.5)	3 (1.8)	0.068 ^b^
POD 3	3 (4.8)	2 (1.2)	0.101^b^
Reintubation rate, number (percentage)	4 (6.5)	2 (1.2)	0.026^b^
Duration of ventilation in minutes, mean (SD)	1,366 (1,843)	439 (357)	≤ 0.001^a^
Intensive care unit length of stay in days, mean (SD)	1.7 (1.4)	0.5 (1.0)	≤ 0.001^a^

^a^Student *t *test; ^b^chi-square test. CK, creatin kinase; CPB, cardiopulmonary bypass; CRP, C-reactive protein; MAP, mean arterial pressure; POD, postoperative day; SD, standard deviation.

### Receiver operating characteristic analysis

**Figure 3:**
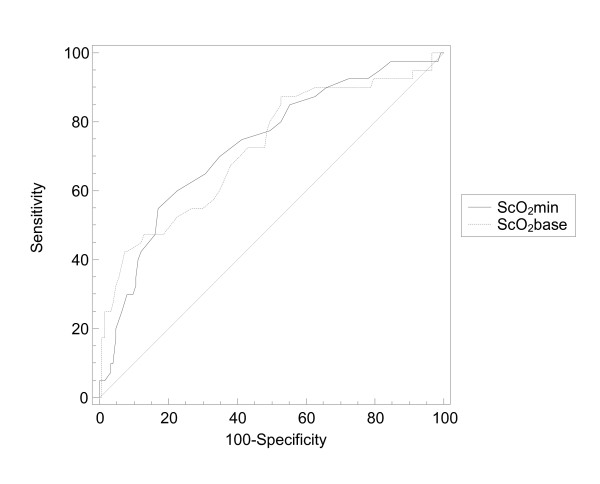
**Comparison between receiver operating characteristic curves for preoperative oxygen-supplemented ScO_2 _and minimal intraoperative ScO_2 _on delirium**. ScO_2_, regional cerebral oxygen saturation; ScO_2_min, minimal intraoperative regional cerebral oxygen saturation; ScO_2_base, regional cerebral oxygen saturation with patient breathing 4 L per minute supplemental oxygen.

The ROC analysis for ScO_2_ox on delirium (AUC 0.72, 95% confidence interval (CI) 0.65 to 0.79, *P *= 0.0001) revealed a best cutoff value of ScO_2_ox of 59.5% (sensitivity 38.2% and specificity 90.3%). The ROC analysis for the minimal intraoperative ScO_2 _(ScO_2_min) on delirium (AUC 0.73, CI 0.66 to 0.80, *P *= 0.0001) revealed a best cutoff of ScO_2_min of 51% (sensitivity 60.0% and specificity 75.6%). Comparison of the two ROC analyses of ScO_2_ox and ScO_2_min showed no significant differences (difference between the areas = 0.01, CI -0.09 to 0.11, *P *= 0.840) as shown in Figure 3.

The ROC analysis for age on delirium (AUC 0.696, *P *= 0.0001) identified a best cutoff age of more than 70 years. The ROC analysis for hemoglobin (AUC 0.635, *P *= 0.003) identified a best cutoff hemoglobin of not more than 120 g/L. ROC analysis for NTproBNP (AUC 0.648, *P *= 0.0001) identified a best cutoff NTproBNP of greater than 995 pg/mL.

### Multivariate analysis

**Figure 4:**
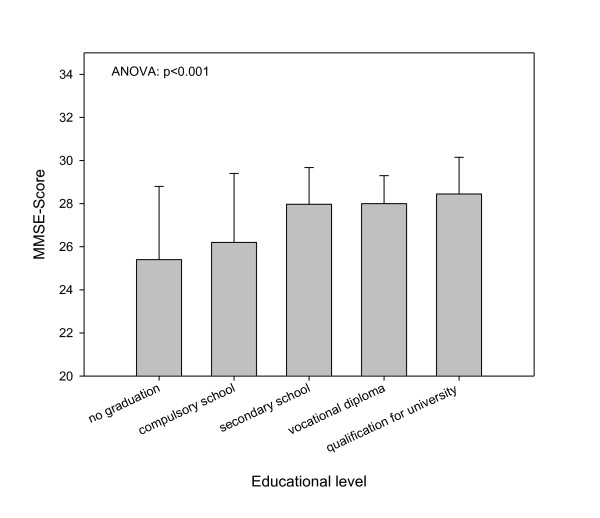
**Mini-Mental Status Examination scores in patients with different educational levels**. ANOVA, analysis of variance; MMSE, Mini-Mental Status Examination.

A multivariate logistic regression was performed with preoperative predictors of delirium which showed significant group differences with a level of significance of less than 1%. As 'educational level' and MMSE were closely related (Figure 4), we decided to include MMSE as a variable for actual cognitive status. MMSE was scaled by clinical criteria. The logistic regression model included the parameters of age, MMSE, additive EuroScore, neurological/psychiatrical disease, preoperative hemoglobin, preoperative NTproBNP, and ScO_2_ox. As the preoperative and the minimal intraoperative ScO_2_min had comparable impact on delirium in the ROC analysis, we decided to choose the preoperative value. In this model (Nagelkerke's R^2 ^= 0.396), age, MMSE, neurological disease, and ScO_2_ox could be identified as independent predictors of delirium (Table 4).

**Table 4:** Binary logistic regression with preoperative predictors of delirium (Nagelkerke's R^2 ^of 39.6%)

Parameter	*P *value	Specification	Prevalence delirium, percentage	Odds ratio	95% CI
Age	0.005	< 70 years	13.0	Reference	
		≥ 70 years	40.5	4.30	1.54-12.04
MMSE score	0.018	> 27	12.2	Reference	
		24-27	27.4	2.23	0.76-6.52
		≤ 23	61.3	6.50	1.75-24.13
Additive EuroScore	0.934	0 to 3	12.1	Reference	
		4 to 6	27.2	0.74	0.19-2.83
		7 to 9	37.1	0.98	0.24-3.95
		10 to 20	40.0	0.77	0.17-3.46
Neurological disease	0.001	No	22.0	Reference	
		Yes	55.2	6.22	2.02-19.16
Hemoglobin	0.513	> 120 g/L	20.4	Reference	
		≤ 120 g/L	47.9	0.72	0.27-1.93
NTproBNP	0.447	< 1,000 pg/mL	17.4	Reference	
		≥ 1,000 pg/mL	41.0	1.43	0.57-3.58
Baseline ScO_2 _with O_2_	0.027	> 59.5%	19.7	Reference	
		≤ 59.5%	58.3	3.27	1.14-9.37

CI, confidence interval; MMSE, Mini-Mental Status Examination; NTproBNP, N-terminal pro B-type natriuretic peptide; O_2_, oxygen; ScO_2_, cerebral oxygen saturation.

## Discussion

The present study reconfirms the known risk factors for postoperative delirium which are old age, altered cognitive function, and neurological or psychiatric disease [4,20,21]. Beyond this, the study identifies preoperative cerebral oxygen saturation (ScO_2_) measured with near-infrared spectroscopy as an important predictor of postoperative delirium. This finding is in line with observations of Morimoto and colleagues [17], who showed a relationship between preoperative ScO_2 _and the incidence of postoperative delirium in a small cohort of non-cardiac surgical patients.

Regarding the technical basis of the method, cerebral oximetry is based on the characteristic of hemoglobin to change its absorbtion spectrum with the degree of oxygenation [22]. Two wavelengths of visible and near-infrared light are used to determine the fraction of oxyhemoglobin in a small region of the frontal lobe. According to the blood distribution in cortical brain tissue, regional cerebral oxygen saturation is a predominantly venous signal and may be regarded as a marker of regional cerebral oxygen balance [15].

Several authors considered relative changes to baseline to be relevant as a threshold for intervention in regional cerebral oxygen saturation [15,23]. In our previous publication, we already had emphasized the meaning of absolute values for outcome prediction [16]. In the present study, we did not find any differences between patients with or without delirium regarding *relative *changes of ScO_2_. Neither between changes from baseline values to minimal intraoperative ScO_2 _nor between the sizes of the AUCs below 80% of the baseline. The *absolute *values did differ and are predictors of delirium. Certainly, absolute values of ScO_2 _are prone to technical influences [24], including sensor location, scattering, and changing path length of the detected light beam [15]. In accordance with our findings, several other studies showed absolute cutoff values that were associated with postoperative cognitive dysfunction after cardiac surgery. Our group showed main effects of intraoperative cerebral desaturation to ScO_2 _of less than 50% on three of four cognitive tests [25], Yao and colleagues [26] showed that an intraoperative ScO_2 _of less than 40% was predictive of postoperative cognitive dysfunction, and Slater and colleagues [27] found an AUC more than 3,000%*sec with cutoff ScO_2 _of less than 50% to be a predictor for cognitive dysfunction. Furthermore, animal studies have shown changes in cellular integrity below absolute cutoff values of ScO_2 _independently of baseline values [28]. Therefore, the absolute ScO_2 _seems to reflect, to a certain degree, the level of cerebral oxygenation impairment. Patients who already start surgery with low ScO_2 _readings can more easily reach deleterious regions of ScO_2 _and therefore are prone to postoperative cerebral impairment.

Interestingly, the present data show that, in patients who start at normal levels of ScO_2_, postoperative delirium is associated with a larger decline in ScO_2_, and this is not the case in patients who already start with marginal ScO_2_. We interpret this finding to be suggestive of the great impact of preoperative ScO_2_. If baseline is already low, the risk of delirium is higher independently of the intraoperative course of ScO_2_. This finding emphasizes the importance of preoperative ScO_2 _measurements. In accordance with several other studies [2,21,29,30], the present study shows that patients who develop delirium after cardiac surgery are older and more severely ill and have lower cognitive capacity than those who did not develop delirium. Kishi and colleagues [24] have already shown a negative correlation between ScO_2 _and age. Impaired cerebral microcirculation in older age and lower oxygen content are possible explanations for this association. Furthermore, an impaired global hemodynamic situation in this more severely ill group of patients might influence the ScO_2 _[16].

Interestingly, neither the hemoglobin level as a parameter contributing to oxygen content nor the NTproBNP as a marker of cardiac insufficiency nor the additive EuroScore could be identified as a predictor of postoperative delirium, leaving old age, cognitive status, neurological disease, and cerebral oxygen saturation contributing to the relative risk.

Carlson and colleagues [31] recently published the interesting association between cognitive function and regional cerebral oxygen saturation in awake older patients. The authors interpreted the ScO_2 _to be a marker of cerebral reserve. Stern [32] has proposed the concept of cognitive reserve to characterize individual differences that allow some people to cope better than others with brain pathology. The cognitive reserve is influenced by both structural and functional factors. In the line with this concept, the ScO_2 _could be understood as a physical marker of cognitive reserve. The low preoperative regional cerebral oxygen saturation in patients who develop postoperative delirium might characterize patients with high susceptibility to cerebral impairment.

Cerebral oxygenation is dependent on arterial oxygen content. To correct a merely hypoxemia-dependent decline in the ScO_2_, we obtained the baseline value on the day before surgery in awake patients after application of supplemental oxygen.

In contrast to our recent findings [16], the present study showed a best predictive value of ScO_2 _of less than 59.5% for postoperative delirium, which is almost 10% higher than the reported ScO_2 _of less than 50% for morbidity and mortality. This discrepancy might be explained, on one hand, by patient selection. The former study included all patients, even those with emergency indication who presented in cardiogenic shock, whereas the present data selected patients for elective or urgent surgery, who were easily able to perform the preoperative minimal cognitive testing. On the other hand, in patients with high susceptibility to cerebral disturbance, an even more subtle set of changes in oxygenation might lead to postoperative delirium. The postoperative findings that patients who develop delirium have greater postoperative morbidity and ventilation time confirm other studies [1].

The present data indicate that preoperative cerebral oxygen saturation influences the risk of postoperative delirium. Further investigation should, therefore, focus on different approaches to optimize preoperative cerebral oxygenation. One pivotal point is the arterial oxygen content. Interestingly, patients who develop delirium had lower preoperative hemoglobin levels. Consistently, they received more transfusions of packed red cells and vasoactive substances. Despite these intraoperative measures to keep oxygen content up to the needed levels, these patients developed delirium more often. Floyd and colleagues [33] depicted an interesting relation between anemia, age, and cerebral blood flow in the context of cardiac surgery. Cerebral oxygenation seems, therefore, to be influenced not only by arterial oxygen content itself but also by secondary cerebral blood flow alterations due to anemia, which is even more pronounced in older patients. Whether preoperative optimization of oxygen content could reduce the risk of delirium and which hemoglobin level should be aimed at need to be subjected to further studies.

Another approach to optimize cerebral oxygenation could be the hemodynamic status. Medium-term application (that is, 12 to 24 hours preoperatively) of positive inotropic substances could possibly increase preoperative cardiac output [34] and consequently cerebral oxygen saturation. The calcium sensitizer levosimendan could be particularly promising as it combines hemodynamic [35], preconditioning [36], and neuroprotective [37] effects.

### Limitations

We did not perform a power analysis for this observational study. However, the CAM-ICU is sufficiently validated to identify delirium in critically ill and cardiac surgical patients [38-40], and the incidence of postoperative delirium of 26% and the proportion of diagnosed hypoactive form of delirium confirm other work in cardiac surgical patients [2,7,39,40].

The latest guidelines for the management of analgesia, sedation, and delirium recommend a monitoring of delirium every 8 hours. Shorter phases of delirium or fluctuating states in the rather large intervals between the measurements might, therefore, have been missed in the present study. Patients who have been categorized as delirious in the present study show a longer duration of ventilation and a longer stay in the intensive care unit. Therefore, the used definition of delirium seems to discriminate a population at risk for a more complex postoperative course.

An important limitation is that the analgesic requirements in the postoperative care were not registered. As the choice as well as the dose of analgesic drugs might trigger delirium [41], it is possible, despite standardized sedation and analgesia protocols, that the incidence of delirium has been biased by postoperative analgesia.

The selection of variables for the multivariate analysis can be discussed. We included the preoperative ScO_2 _but not the minimal intraoperative value. This decision was based on the intention to identify predictive parameters for postoperative delirium.

The inclusion of MMSE but not educational level in the multivariate model might be questioned. The close relation between the MMSE and the educational level would have confounded the model. However, we decided that the actual cognitive status might be more representative than educational level, which might be influenced by various demographic factors. The EuroScore compiles variables of cardiac, pulmonary, and renal function as well as extracardiac vascular pathology and demographic factors like age. Because age is such a relevant predictor for postoperative delirium [2,6,42], we accepted the inclusion of age as a separate variable as well as a contributor to the additive EuroScore. This decision might have led to an over-representation of age in the model. The factor of neurological disease included psychiatric disorders (that is, depression) as well as stroke or mild dementia. All of these diseases have in common a possibly high sensitivity to hemodynamic, oxygenative, or metabolic disturbances [43]. Nonetheless, the information given by this composite variable is certainly questionable.

## Conclusions

The present study identifies the preoperative cerebral oxygen saturation as well as age, cognitive status, and neurological or psychiatric disease as independent predictors of postoperative delirium in cardiac surgical patients. The association of the cerebral oxygen saturation to functional aspects of cerebral impairment (that is, altered cognitive function) as well as structural aspects of cerebral integrity (that is, old age or hemodynamic condition) makes it an interesting tool for predicting and possibly preventing cognitive disturbances after cardiac surgery.

## Key messages

• Patients who develop postoperative delirium after cardiac surgery have a lower preoperative cerebral oxygen saturation.

• The relative intraoperative decline in cerebral oxygen saturation does not differ in patients with and without delirium.

• Along with the known preoperative risk factors of age, cognitive status, and neurological disease, preoperative regional cerebral oxygen saturation is an independent predictor of postoperative delirium.

## Abbreviations

AUC: area under the curve; CAM-ICU: Confusion Assessment Method for the Intensive Care Unit; CI: confidence interval; MMSE: Mini-Mental Status Examination; NTproBNP: N-terminal pro B-type natriuretic peptide; RASS: Richmond Agitation Sedation Scale; ROC: receiver operating characteristic; SaO_2_: arterial oxygen saturation; ScO_2_: regional cerebral oxygen saturation; ScO_2_min: minimal intraoperative regional cerebral oxygen saturation; ScO_2_ox: regional cerebral oxygen saturation after compensation of hypoxemia; ScO_2_room: regional cerebral oxygen saturation obtained in a sitting position without supplemental oxygen.

## Competing interests

JS and MHe receive honoraria for lectures from Covidien Germany GmbH (Neustadt, Germany). The other authors declare that they have no competing interests.

## Authors' contributions

JS participated in conduction of the study, analyzed the data, and wrote the manuscript. JM conducted the cognitive testing and helped to write the manuscript. HP participated in the conduction of the study. MH participated in the design of the study, conduction of the study, and writing the manuscript. MH participated in the design of the study and conduction of the cognitive testing and analyzed the data. K-UB designed the study and participated in the conduction of the study and in writing the manuscript. All authors read and approved the final manuscript.
